# Effect of cryotherapy in preventing mucositis associated with the use of 5-fluorouracil

**DOI:** 10.1590/1518-8345.3953.3363

**Published:** 2020-10-19

**Authors:** Andrea Bezerra Rodrigues, Maria Isis Freire De Aguiar, Patrícia Peres De Oliveira, Naiana Pacifico Alves, Renan Alves Silva, Willame De Oliveira Vitorino, Thays Silva De Souza Lopes

**Affiliations:** 1 Universidade Federal do Ceará, Departamento de Enfermagem, Fortaleza, CE, Brazil.; 2 Universidade Federal de São João Del Rey, Departamento de Enfermagem, Divinópolis, MG, Brazil.; 3 Universidade Federal do Espírito Santo, Departamento de Ciências da Saúde, São Mateus, ES, Brazil.

**Keywords:** Cryotherapy, Stomatitis, Fluorouracil, Antineoplastic Agents, Evidence-Based Nursing, Nursing, Crioterapia, Mucosite Oral, Fluorouracil, Antineoplásicos, Enfermagem Baseada em Evidências, Enfermagem, Crioterapia, Estomatitis, Fluorouracilo, Antineoplásicos, Enfermería Basada en la Evidencia, Enfermería

## Abstract

**Objective:**

to evaluate the effect of oral cryotherapy compared to physiological serum on the development of oral mucositis in outpatient cancer patients using the 5-fluorouracil antineoplastic agent.

**Method:**

this is a controlled, randomized, double-blind, and multi-center clinical trial, conducted with 60 patients undergoing chemotherapy. The experimental group (n=30) used oral cryotherapy during the infusion of the 5-FU antineoplastic agent, while the control group (n=30) performed mouthwash with physiological serum at their homes. The oral cavity of the participants was assessed at three times: before randomization, and on the 7^th^ and 14^th^ days after using 5-FU. For data analysis, descriptive analyses and the ANOVA, paired t, and McNemar tests were used.

**Results:**

there was no statistically significant difference between the experimental and control groups in the assessments regarding the grade of mucositis. However, cryotherapy presented the chance to reduce the presence of intragroup mucositis, between the first and second assessments (p=0.000126).

**Conclusion:**

cryotherapy did not obtain statistical significance in relation to oral hygiene with serum, but it proved to be effective intragroup*.* Record number: RBR-4k7zh3

## Abstract (pt)

**Objetivo:**

avaliar o efeito da crioterapia oral em comparação ao soro fisiológico no desenvolvimento de mucosite oral em pacientes oncológicos ambulatoriais em uso de antineoplásico 5-fluorouracil.

**Método:**

trata-se de um ensaio clínico controlado, randomizado, duplo-cego, multicêntrico, realizado com 60 pacientes em tratamento quimioterápico. O grupo experimental (n=30) fez uso de crioterapia oral durante a infusão do antineoplásico 5-FU, ao passo que o grupo controle (n=30) realizou bochechos com soro fisiológico em domicílio. A cavidade oral dos participantes foi avaliada em três momentos: antes da randomização, no 7° dia e no 14° dia após o uso do 5-FU. Para a análise dos dados, utilizaram-se análises descritivas e os testes ANOVA, t pareado e McNemar.

**Resultados:**

não houve diferença estatística significante entre os grupos experimental e controle nas avaliações quanto ao grau de mucosite. No entanto, a crioterapia teve a chance de reduzir a presença de mucosite intragrupo, entre a primeira e segunda avaliação (p=0,000126).

**Conclusão:**

a crioterapia não obteve significância estatística em relação à higiene oral com soro, porém se mostrou efetiva intragrupo*.* Número de registro: RBR-4k7zh3

## Abstract (es)

**Objetivo:**

evaluar el efecto de la crioterapia oral, comparándola con el suero fisiológico, sobre el desarrollo de mucositis oral en pacientes ambulatorios con cáncer en tratamiento con el antineoplásico 5-fluorouracilo.

**Método:**

se trata de un ensayo clínico controlado, aleatorizado, a doble ciego y multicéntrico, realizado con 60 pacientes en tratamiento de quimioterapia. El grupo experimental (n=30) utilizó crioterapia oral durante la administración del agente antineoplásico 5-FU, mientras que el grupo control (n=30) realizó enjuagues con suero fisiológico en su domicilio. La cavidad oral de los participantes se evaluó en tres momentos: antes de la aleatorización, y al 7º y 14º día después de la administración de 5-FU. Para el análisis de los datos, se utilizaron análisis descriptivos y las pruebas ANOVA, t pareada y McNemar.

**Resultados:**

en lo que respecta al grado de mucositis, no se registraron diferencias estadísticamente significativas entre el grupo experimental y el de control en las evaluaciones. Sin embargo, la crioterapia exhibió la posibilidad de reducir la presencia de mucositis intragrupo entre la primera y la segunda evaluación (p=0,000126).

**Conclusión:**

la crioterapia no alcanzó significancia estadística en comparación con la higiene oral con suero. Sin embargo, demostró ser efectiva intragrupo. Número de registro: RBR-4k7zh3

## Introduction

Oral Mucositis (OM) is a complex biological process. The pathogenesis of OM comprises a sequence of biological events possibly influenced by the oral microbiome and by the environment, leading to the positive regulation of pro-inflammatory cytokines, resulting in the thinning of the epithelium through tissue damage and cell death^(^ 1 ^)^.

People receiving chemotherapy for cancer are at risk of developing mucositis as a side effect^(^ 2 ^)^, which can occur in 20% to 40% of the patients undergoing conventional chemotherapy regimens^(^ 3 ^)^.

Among the antineoplastic agents that cause these changes in the oral mucosa is 5-fluorouracil (5-FU)^(^ 4 ^)^. 5-FU is a drug used in the treatment of solid cancers, such as those of the gastrointestinal tract and breast, and has a short half-life^(^ 5 ^)^.

OM is a side effect that causes several changes in the patient, such as pain, difficulty in eating, risk of infection and bleeding, and distress, in addition to causing an increase in the treatment cost, both for the patient and for the health system, since it needs medications to control pain and infections and often requires hospitalization for enteral support^(^ 1 ^-^ 6 ^)^.

Oral cryotherapy is characterized by the application of ice in the oral cavity or by mouth rinsing with iced water before, during, and after the administration of the chemotherapy drugs^(^ 7 ^)^. The use of cryotherapy is based on the assumption that ice-induced vasoconstriction will reduce blood flow in the oral mucosa, resulting in lower local concentrations of the chemotherapeutic agents, reducing the chance of OM^(^ 1 ^)^.

Therefore, the objective of this study was to verify the effect of cryotherapy compared to the oral hygiene protocol with physiological serum in reducing the incidence and severity of oral mucositis in patients using 5-fluorouracil in *bolus*.

## Method

A controlled, randomized, double-blind, and multi-center clinical trial, carried out between December 2016 and December 2018 in two Brazilian cancer chemotherapy outpatient clinics belonging to the Unified Health System (*Sistema* Único *de Saúde*, SUS).

The study population consisted of cancer patients admitted to the aforementioned chemotherapy outpatient clinics. To establish the sample size, it was estimated based on a previous study^(^ 8 ^)^, where the power calculation to determine the number of participants in each group was performed in relation to the expected change in the grade of mucositis, as assessed by the World Health Organization Scale^(^ 9 ^)^. Thus, considering a level of p<0.05 and 80% of power as significant, a minimum of 24 participants in each group was necessary. However, 30 participants were included in each group, corresponding to a total of 60 participants.

A consecutive sample of patients who attended outpatient chemotherapy units was assessed for eligibility. The inclusion criteria were the following: patients over 18 years of age, with some solid cancer, undergoing therapy with 5-fluorouracil (5-FU) in *bolus* as part of the chemotherapy protocol, regardless of the protocol used. Patients undergoing treatment with 5-FU who had any of the following criteria were excluded from the study: radiotherapy treatment in the head and neck region, smoking and alcoholism habits, history of tooth sensitivity, use of oxaliplatin chemotherapy concomitant with the use of 5-FU, since cryotherapy tends to aggravate the neurotoxicity caused by this chemotherapy.

### Study design

Before the start of collection, the researchers introduced themselves to the nursing staff in the field of study and explained the study, highlighting the criteria for inclusion and exclusion of patients in the research.

Two assistant researchers were trained and instructed not to provide information to any participant about the use of cryotherapy and physiological serum to prevent mucositis, as well as guidelines on oral hygiene, adequate nutrition or others that could interfere with the results of the research.

On the collection days, the nurses signaled the patients considered eligible to the two assistant researchers. In the first moment, the first assistant researcher provided the patient with all the information regarding the study. The patients were informed about the need for new assessments after the intervention and that one of the researchers would contact them by phone to schedule a place and time for the subsequent evaluations, which could take place at the outpatient clinic or at their homes, according to their preference. After the guidelines, the patients were invited to participate in the research and, if they agreed, the signing of the Free and Informed Consent Form (FICF) and the assessment of the grade of mucositis before the intervention were performed.

In the second moment, the second assistant researcher took out an envelope previously prepared revealing in which group the patient would be included and applied the intervention. The sealed and opaque envelopes, sequenced with the “experimental group” (EG) or “control group” (CG) designation, were made by a professional with no involvement in data collection. It should be noted that the researchers responsible for the application of the data collection instrument were not involved in the application of the intervention.

The patients were randomized by a statistician into two groups: experimental group (EG) and control group (CG), with a 1:1 allocation rate, using a table of random numbers generated in the Epi Info software, version 7.1.4.

The person responsible for the statistical analysis was also blinded since, before the data were made available, the CG and EG were coded in G1 and G2 to prevent him from distinguishing the group that received the intervention.

Clinical and sociodemographic data, such as age, schooling, marital status, type of cancer, and chemotherapy treatment protocol were collected from the patient’s medical record; information about origin, current occupation, income, religion, smoking and alcoholism habits, use of dental prosthesis, frequency of dental consultations, and use of mouthwashes were collected from interviews with the participant, using an instrument developed by the researchers.

For the participants in the experimental group, the researcher applied ice to the oral cavity, starting 5 minutes before the 5-FU infusion and lasting for 30 minutes of continuous administration, as recommended^(^ 10 ^)^. It should be noted that there is no determination in the literature about the amount of grams of ice to be applied, only that the ice should be easily moved in the oral cavity, for which ice pieces are recommended. Therefore, the participant was provided with pieces of ice packaged in a plastic cup for individual use and a napkin. The ice was being replaced as it ran out. For the patients with dental prostheses, their removal and packaging in a plastic cup was requested before the intervention.

The participants in the control group were instructed to perform mouthwashes with 10 ml of physiological serum, at room temperature, three times a day, for one minute, and for a period of 14 days after application of the chemotherapy drug. All the instructions for the use of physiological serum were inserted in a label that was attached to each serum bottle provided by the researchers to the patient.

*Measurement of mucositis:* mucositis was evaluated for both experimental and control groups, according to the World Health Organization (WHO) mucositis assessment scale^(^ 9 ^)^, a scale considered ideal because it incorporates both clinical and functional factors, and has its validity established in several studies on the theme^(^ 8 ^,^ 11 ^-^ 13 ^)^. On this scale, mucositis is classified into four grades, namely: Grade 0: no changes; Grade 1: erythema; with or without pain; Grade 2: erythema, ulcers, the patient can eat a solid diet; Grade 3: ulcers, extensive erythema, the patient is unable to eat a solid diet; Grade 4: extensive mucositis that does not allow oral feeding. To assess mucositis, personal protective equipment was used, in addition to a pocket flashlight.

The outcome was measured in three moments. The first evaluation was carried out before the administration of 5-FU (T1), the second evaluation 7 days after intervention (T2), and the third, 14 days after (T3). These evaluation points were established considering that the symptoms of mucositis develop from the 5^th^ day after the administration of the chemotherapy drug in question, and may last until the 14^th^ day^(^ 3 ^)^.

**Figure 1:**
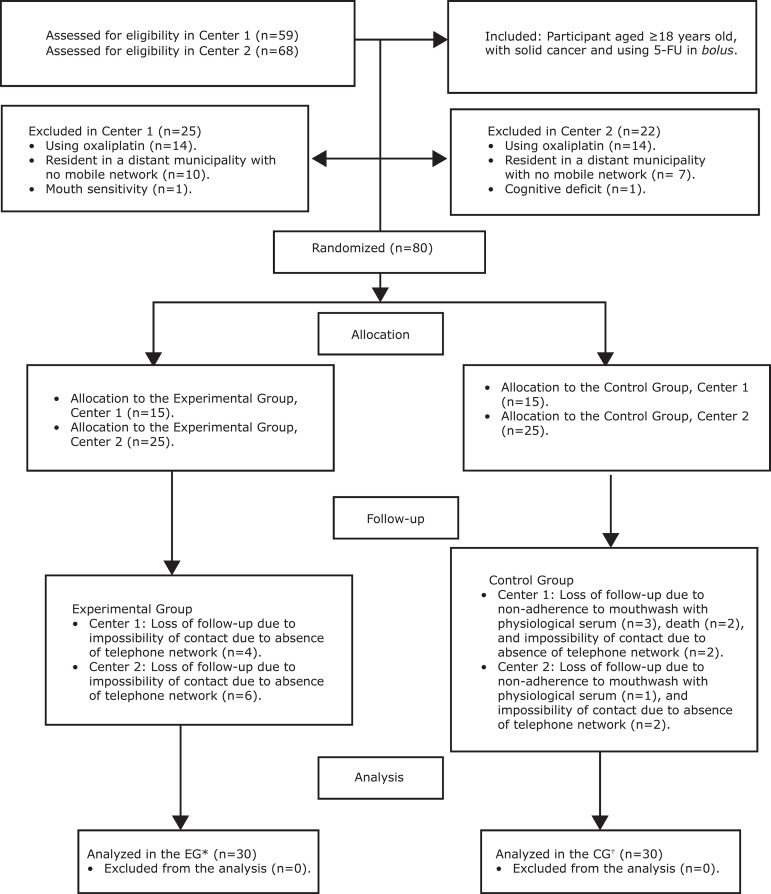
Flow diagram of the study. Fortaleza, CE, Brazil, 2018 ^*^EG = Experimental Group; ^†^CG = Control Group

A total of 127 participants were assessed for eligibility (Figure 1) at the two centers where the research was conducted. Of these, 25 and 22 did not meet the inclusion criteria in Centers 1 and 2, respectively. Thus, 80 participants were randomized in the clinics between December 2016 and December 2018. There were ten losses in the follow-up of the experimental group due to the impossibility of contact by phone, 4 in Center 1 and 6 in Center 2, and ten losses in follow-up in the control group due to the participant’s non-adherence to mouthwash with physiological serum during the 14 days (4 patients), death (2 patients) or impossibility of contact by phone (4 patients), totaling 7 at Center 1, and 3 at Center 2. There were no analysis losses. Thus, the final sample consisted of 30 patients in each group.

The data obtained from the research instruments were entered twice and organized in an electronic spreadsheet using the Microsoft Office Excel 2007 program. Subsequently, this spreadsheet was exported to the IBM - SPSS statistical program, version 22, in which all the statistical analyses of this study were carried out. The variables for sociodemographic characterization of the sample were analyzed using descriptive statistics, with analyses of distributions and frequencies. In order to verify homogeneity between the control and experimental groups, Levene’s homogeneity test was applied.

The McNemar test was performed to assess the significance of intragroup changes, in different assessments of the grade of mucositis. In this test, each participant is used as their own control, and the measurement is made on a nominal or ordinal scale. For the intervention to show significance of change, the results obtained must be greater than 1, and their respective confidence intervals must not exceed the null value, which is 1. The significance level of the statistical test was set at 5% (ɑ=0.05).

To analyze the intergroup comparison, the ANOVA test was used, with a significance level of 5% (ɑ=0.05).

The research was approved by the Research Ethics Committees of the Federal University of Ceará and of the Walter Cantídio University Hospital (CAAE: 57369316.9.00005054), recorded in the Brazilian Registry of Clinical Trials platform (RBR-4k7zh3).

## Results

The sample consisted equally of men (n=30) and women (n=30) in the groups, with a mean age of 56.15 (±14.95), and the majority having gastrointestinal tract cancer, both in the EG (83.3%) and in the CG (93.3%). The sample variables that showed homogeneity were the following: age (p=0.152), gender (p=0.849), occupation (p=0.597), schooling (p= 0.791), family income (p=0.220), religion (p=0.157), smoking (p=0.053), alcoholism (p=0.874), use of dental prosthesis (p=0.453), daily oral hygiene (p=0.453), use of mouthwash (p=0.254), and periodic visit to the dentist (p=0.675). There was no homogeneity regarding the type of cancer (p=0.005).

Both the patients in the CG and in the EG did not have a regular habit of using mouthwash, the same occurring with respect to regular visit to the dentist in the vast majority of the sample of patients in the CG (76.6%) and in the EG (70.0%) (Table 1).

**Table 1:** Demographic and clinical characterization of the participants in the control and experimental groups. Fortaleza, CE, Brazil, 2018

Variables		Group		
		Control	Experimental	P
Gender	Male	15 (50%)	15 (50%)	1.00*
	Female	15 (50%)	15 (50%)	
				
Origin	Capital	25 (83.3%)	15 (50%)	0.013*
	Inland	5 (16.7%)	15 (50%)	
Occupation	Domestic worker	1 (3.3%)	2(6.65%)	0.989^†^
	Housewife	1 (3.3%)	2 (6.65%)	
	Autonomous professional	4 (13.4%)	4(13.4%)	
	Public server	1 (3.3%)	1 (3.3%)	
	Retired	9 (30%)	9(30%)	
	Other	14 (46.7%)	12 (40%)	
Schooling	Illiterate	4 (13.4%)	7(23.3%)	0.854^†^
	Incomplete elementary school	13 (43.3%)	10 (33.34%)	
	Complete elementary school	4(13.4%)	3(10%)	
	Incomplete high school	0 (0%)	1(3.3%)	
	Complete high school	6 (20%)	6 (20%)	
	Higher education	3 (10%)	3 (10%)	
Marital status	Single	11 (36.7%)	5(16.7%)	0.214^†^
	Married	14 (46.7%)	15 (50%)	
	Stable union	2 (6.65%)	4 (13.3%)	
	Widow	1 (3.3%)	5 (16.7%)	
	Divorced	2 (6.65%)	1 (3.3%)	
Family income	Less than 1 minimum wage	9 (30%)	7 (23.3%)	0.758^†^
	1 to 3 minimum wages	19 (63.35%)	22 (73.4%)	
	>3 to <=7 minimum wages	2 (6.65%)	1 (3.3%)	
Religion	Catholic	22 (73.3%)	24 (80%)	0.552^†^
	Evangelical	8 (26.7%)	6 (20%)	
	Others	1 (3.3%)	0 (0%)	
Smoking	Yes	11 (36.7%)	8 (26.7%)	0.580*
	No	19 (63.3%)	22 (73.3%)	
Dental prosthesis	Yes	19 (63.3%)	14 (46.7%)	0.299*
	No	11 (36.7%)	16 (53.3%)	
Mouthwash	Yes	5 (16.7%)	8 (26.7%)	0.532*
	No	25 (83.3%)	22 (73.3%)	
Visit to the dentist	Yes	7 (23.3%)	9 (30%)	0.771*
	No	23 (76.7%)	21 (70%)	
Alcoholism	Yes	7 (23.3%)	7 (23.3%)	1.00*
	No	23 (76.7%)	23 (76.7%)	
Type of cancer	Genitourinary tract	0 (0%)	3 (10%)	0.025^†^
	Gastrointestinal tract	25 (83.35%)	27 (90%)	
	Breast	2 (6.65%)	0 (0%)	
	Other tumors	3 (10%)	0 (0%)	
Comorbidities	Yes	10 (33.3%)	15 (50%)	0.295*
	No	20 (66.7%)	15 (50%)	
Diabetes Mellitus	Yes	5 (16.7%)	9 (30%)	1.00*
	No	25 (83.3%)	21 (70%)	
Systemic Arterial Hypertension	Yes	5 (16.7%)	7 (33.3%)	1.000*
	No	25 (83.3%)	23 (76.7%)	
Total		30 (100%)	30 (100%)	

* Chi-square test;

† Fisher's exact test

Regarding the clinical protocols for chemotherapy treatment, it was verified that 48.33% used Flox (5-FU+oxaliplatin) and 5-FU+leucovorin (Mayo Clinic) (20%). It is noteworthy that cryotherapy was applied to the participants who received the Flox protocol only on the days when they did not receive oxaliplatin. Separating by group, we have that, in the EG, 40% used Flox, while 23.3% received 5-FU+leucovorin. In the CG, 36.67% used Flox, followed by 16.67% of 5-FU+leucovorin. There was homogeneity between the groups in relation to the clinical protocol (p=0.889).

Regarding the interval between cycles, it was identified that both established protocols had a weekly interval. It was verified that there was also homogeneity between the cycle intervals (p=0.076).

There was a significant variation in the prevalence of the grade of mucositis outcome in both groups at moments T1 and T2. At T3, there was a slight reduction in relation to T2, in both groups. Even with this similar delineation of the occurrence of mucositis in both groups, it is observed that the CG showed peak prevalence at T2 (26.6%).

Regarding the presence of mucositis, in both groups, it is verified that, at T1, 10% of all the patients presented mucositis, which increased to 21.7% at T2, decreasing to 8.3% at T3.

Considering separately by group, it is observed that the patients of the EG had a higher prevalence of mucositis at T1, when compared to the CG (13.3% x 6.7%). At T2, there is 26.6% prevalence of mucositis in the CG, while in the EG the prevalence of the outcome was 13.3%. It is observed that, at that moment, the prevalence of mucositis in the patients selected in the EG was maintained in relation to the result of the previous evaluation. In relation to the CG, it is verified that the prevalence of mucositis doubles in relation to the previous assessment. Regarding T3, it was verified that the prevalence of mucositis in the CG was 13.3% while in the EG it was 3.3%. It is also seen that the absence of Grade 2 mucositis in the EG was prevalent (Table 2).

**Table 2:** Distribution of the grades of mucositis outcome of the participants at different assessment moments, in the control and experimental groups. Fortaleza, CE, Brazil, 2018

Assessments	Grades*	Control	Experimental	Total
T1	Grade 0	28	26	54
		93.3%	86.7%	90.0%
	Grade 1	2	3	5
		6.7%	10.0%	8.3%
	Grade 2	0	1	1
		0.0%	3.3%	1.7%
T2	Grade 0	23	26	47
		76.7%	86.7%	78.3%
	Grade 1	7	4	12
		23.3%	13.3%	20.0%
	Grade 2	1	0	1
		3.3%	0.0%	1.7%
T3	Grade 0	26	29	55
		86.7%	96.7%	91.7%
	Grade 1	4	1	5
		13.3%	3.3%	8.3%
	Grade 2	0	0	0
		0%	0%	0%
Total		30	30	60
		100.0%	100.0%	100.0%

* According to the scale of the World Health Organization

When analyzing the intergroup comparison, it was verified that cryotherapy showed limited efficacy, since there was no statistically significant difference between the experimental and control groups in the different assessments regarding the grade of mucositis (Table 3).

**Table 3:** Interclass analysis of the mucositis outcome at the different assessment moments. Fortaleza, CE, Brazil, 2018

Assessments	Mean		P*	S^†^	ANOVA
	EG^‡^	CG^§^			
T1^||^	0.1667	0.0667	0.3859	1.083	0.302
			0.3447		
T2^¶^	0.1667	0.3000	0.8201	0.0000	1.000
			1.000		
T3**	0.0330	0.1334	0.1694	1.962	0.167
			0.2330		

* Value of the test;

† Source of variance;

‡ Experimental Group;

§ Control Group;

|| First mucositis assessment;

¶ Second mucositis assessment;

** Third mucositis assessment

Regarding the reduction of mucositis in the EG (intraclass analysis), it was verified that cryotherapy had a chance of approximately six times (OR: 6.5; X^2^=14.7; DoF:1; CI: 2.68 -201.99) to reduce the presence of mucositis regardless of the grade in the individuals selected between the first and the second assessments, presenting significant statistics (p=0.000126). Between the second and third assessments, and between the first and third, the use of this intervention was able to reduce by approximately seven times the chance of presenting mucositis, in each comparison (OR: 7.25; X^2^=17.4; DoF:1; CI: 3.03-189.91).

## Discussion

In the present study, there was no statistically significant difference between the groups that used cryotherapy or saline solution. However, cryotherapy had the chance to reduce the presence of intragroup mucositis, between the first and second assessments (p=0.000126), which may support the use of oral cryotherapy to prevent and control OM.

Similarly to this study, a randomized clinical trial conducted with 80 patients with colorectal cancer treated with the same 5-FU drug verified that the patients who received oral cryotherapy together with mouthwash were less likely to report the occurrence of mucositis, compared to usual care patients who used only mouthwash^(^ 14 ^)^.

Cryotherapy is effective in preventing oral mucositis in patients scheduled for chemotherapy with antineoplastic agents with short plasma half-lives, such as *bolus* doses of 5-fluorouracil^(^ 14 ^)^. A clinical trial that investigated the effects of oral cryotherapy on chemotherapy-induced oral mucositis in patients undergoing autologous transplantation verified that cryotherapy is more effective than saline mouthwash in reducing the severity of mucositis^(^ 6 ^)^ and, in this sense, a systematic review by Cochrane reported evidence showing that oral cryotherapy can lead to large reductions in the number of adults reporting oral mucositis of all severities after receiving a fluorouracil-based treatment for solid neoplastic malignancies^(^ 2 ^)^.

Another study, a clinical trial that used oral cryotherapy for 58 patients with esophageal cancer using the DCF (docetaxel, cisplatin and fluorouracil) chemotherapy protocol, verified the reduction of the incidence of oral mucositis of all grades in comparison with the non-cryotherapy group (24.1% x 71.4%, p<0.001)^(^ 15 ^)^. A meta-analysis that included 14 studies with 1,280 participants also concluded that oral cryotherapy reduced the risk of developing mucositis in patients treated with fluorouracil-based chemotherapy^(^ 16 ^)^.

These findings, including that of this study, give credibility to the use of oral cryotherapy as a low cost prophylactic measure against oral mucositis in patients with malignant neoplasia undergoing chemotherapy based on 5-fluorouracil, and oral cryotherapy reduces the risk of developing mucositis in patients treated with 5-FU-based chemotherapy.

With regard to mouthwash solutions, the use of chlorhexidine, sodium bicarbonate, benzydamide, and saline solution was identified^(^ 5 ^)^. A clinical trial aimed at health education on oral hygiene, including mouthwash with salt water with glutamine^(^ 17 ^)^ found no difference between the results regarding OM with the different solutions.

Thus, the scientific evidence points to the effectiveness of interventions such as oral hygiene protocols, which was intended to be tested in comparison to cryotherapy in this study. More than the mouthwash solution itself, the unique component of any oral hygiene protocol for reducing OM is use consistency, which has a positive effect, both in prevention and reduction^(^ 17 ^)^.

When the effectiveness of intra-group cryotherapy was evaluated, this intervention had the chance to reduce by approximately six times the presence of mucositis between the first and the second evaluations, regardless of the grade, presenting significant statistics. Between the second and third assessments, and between the first and the third, the use of this intervention was able to reduce by approximately seven times the chance of presenting mucositis.

Another relevant fact was that the experimental group did not present higher grades of mucositis in any of the evaluations, differently from the control group. This fact was also verified in other studies^(^ 8 ^,^ 11 ^)^, showing that cryotherapy can, in addition to reducing the onset of mucositis, prevent the occurrence of higher grades of this outcome.

A meta-analysis of 29 clinical trials that evaluated, among other interventions, cryotherapy for the prevention of oral mucositis induced by chemotherapy in adult cancer patients, states that it was the most effective intervention in the prevention of OM, with a safety profile similar to the control^(^ 18 ^)^.

A larger number of studies using cryotherapy in a population of patients with onco-hematological disease was identified than in solid tumors, as in the present study. Onco-hematological patients can be subjected to induction for hematopoietic stem cell transplantation with another drug, melphalan, also a potential etiologic factor for mucositis^(^ 7 ^,^ 19 ^-^ 21 ^)^.

Thus, the measures currently available for the prevention and control of OM, excluding cryotherapy and oral hygiene protocols, are expensive, such as laser therapy, which has a proven indication only for patients undergoing hematopoietic stem cell transplantation and patients with head and neck cancer undergoing chemo or radiation therapy^(^ 3 ^)^.

In this sense, the cryotherapy intervention is seen as capable of being applied by nurses, as a low cost and easy application method, without adverse effects, mainly considering that the chemotherapy protocols with the use of 5-FU in *bolus* are performed on an outpatient basis. Parallelly, the participants had a good acceptance of the intervention, since there were no follow-up losses in the experimental group regarding difficulties in its use.

The results of this study support the continuation of a recommendation for the use of cryotherapy to prevent oral mucositis in patients receiving chemotherapy with 5-fluorouracil, as stated in the evidence-based clinical practice guidelines of MASCC/ISOO^(^ 10 ^)^ for mucositis. The cryotherapy protocol included the administration of pieces of ice starting 5 minutes before the 5-FU infusion and lasting for 30 minutes during chemotherapy. No major side effects have been reported. Nurses have an important role in informing the patients about the benefits of cryotherapy and supporting them during the administration of chemotherapy and the application of cryotherapy.

The following are highlighted as limitations in this study: measuring the outcome by only one cycle of chemotherapy, and the difficulty in controlling the lifestyle habits of the participants, such as food and oral hygiene, can interfere in the mucositis outcome. Therefore, the results cannot be generalized to all the patients receiving the 5-fluorouracil antineoplastic agent.

It is suggested to conduct research studies that involve more participants, in order to provide more accurate findings and that evaluate oral hygiene protocols with the use of saline solution, still incipient in research.

## Conclusion

Although cryotherapy did not obtain statistical significance, when compared to the oral hygiene protocol with saline solution, it proved to be effective intragroup*.* The inclusion of cryotherapy on an outpatient basis for patients undergoing treatment with the 5-FU chemotherapy drug can be an alternative to reduce the occurrence and severity of mucositis. The results of this study help to clarify evidence that supports the use of oral cryotherapy, which is economical and has few side effects, as a preventive strategy for oral mucositis.
